# Supplementary material for: Clinical implications of Type 2 diabetes on outcomes after cardiac transplantation
Source: PLoS One. 2022 Dec 14;17(12):e0273111. doi: 10.1371/journal.pone.0273111 (PMC9750001; doi:10.1371/journal.pone.0273111)
Supplement: S1 File — (DOCX) [file pone.0273111.s001.docx]

**Supplementary Data/Supporting Information**

**Legend:**

**S1 Fig.** A) Listing Status by allocation system, in patients without T2D, B) Transplantation Status by allocation system, in patients with T2D

**S2 Fig.** A) 1-Year Post Transplant Mortality by Year of Transplant, B) 2-Year Post Transplant Mortality by Year of Transplant, C) 3-Year Post Transplant Mortality by Year of Transplant

**S3 Fig.** 1-Year Waitlist Mortality by Year of Listing

**S1 Fig.** A) Listing Status by allocation system, in patients without T2D


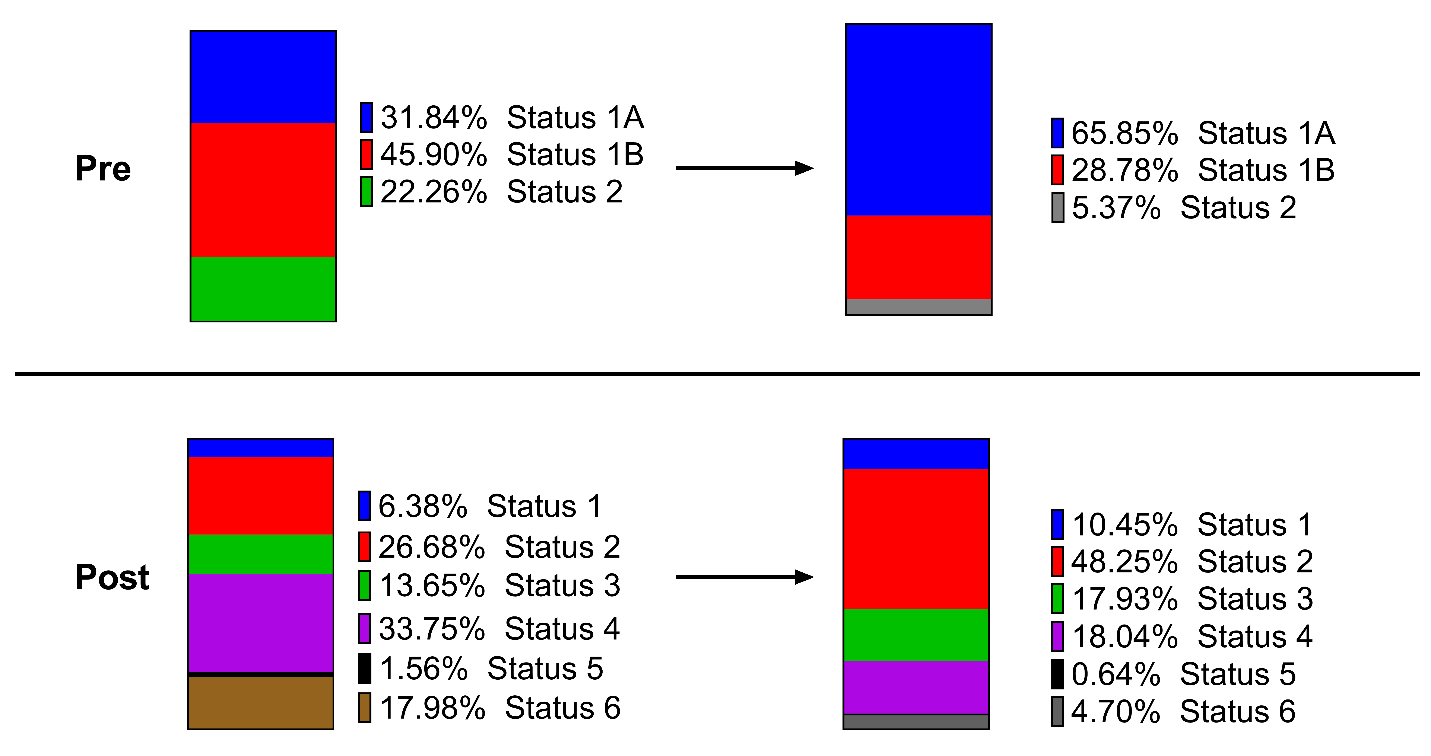


B) Transplantation Status by allocation system, in patients with T2D


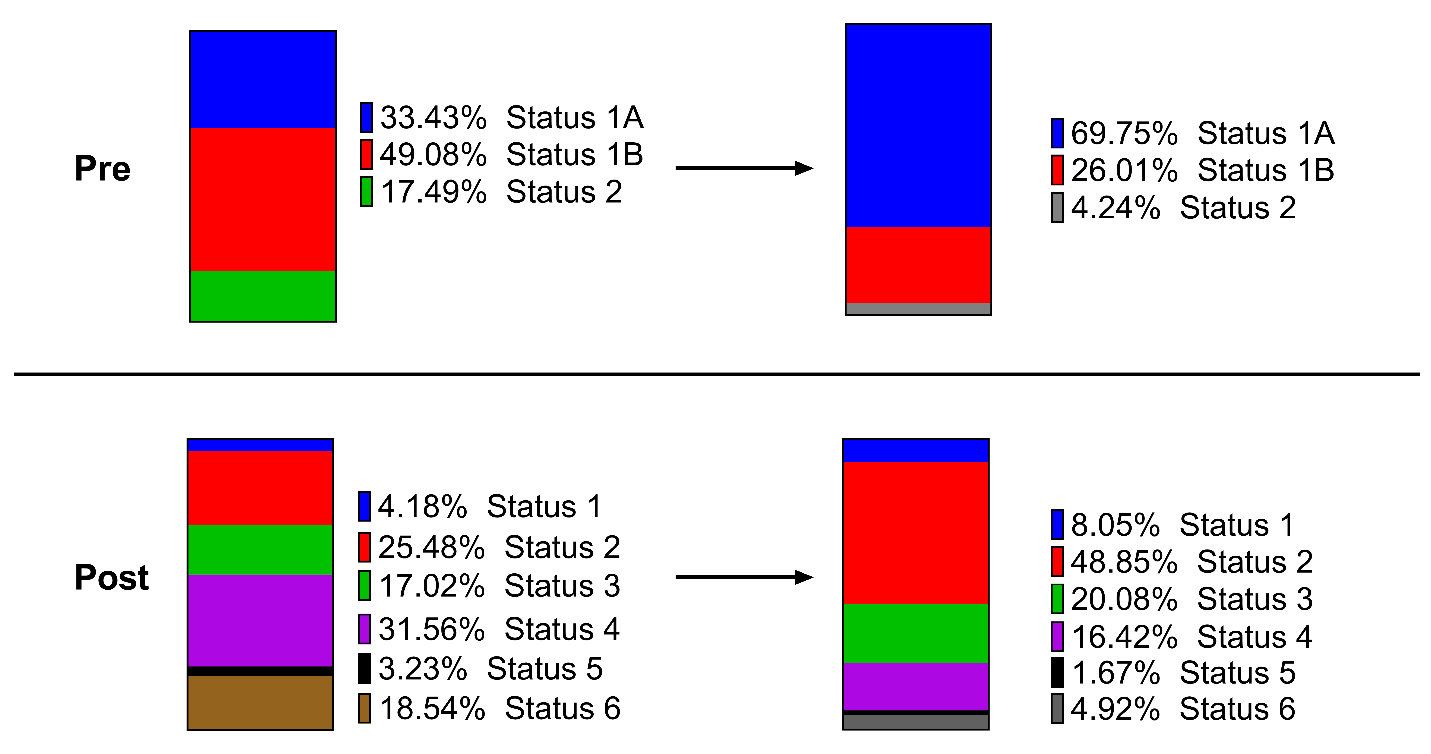


**S2 Fig.** A) 1-Year Post Transplant Mortality by Year of Transplant

B) 2-Year Post Transplant Mortality by Year of Transplant

C). 3-Year Post Transplant Mortality by Year of Transplant

**S3 Fig.** 1-Year Waitlist Mortality by Year of Listing
