# Supplementary material for: Clinical implications of Type 2 diabetes on outcomes after cardiac transplantation
Source: PLoS One. 2022 Dec 14;17(12):e0273111. doi: 10.1371/journal.pone.0273111 (PMC9750001; doi:10.1371/journal.pone.0273111)
Supplement: Attachment — Submitted filename: Plos One Diabetes Response to Reviewers_Final_Final.docx [file pone.0273111.s003.docx]

**Response to Reviewers:**

Editor:

1. The authors unfortunately haven't fully addressed the issues raised by the reviewers in the MS itself, and have decided to provide the requested information in the supplements. Donor age is a crucial prognostic factor in hear transplantation, and has not been analysed in multivariate analysis. These crucial changes have to be made in the MS in order for the MS to be reconsidered.

**Author Response: Thank you for these important recommendations. We agree that it is important to highlight the issues brought up by reviewer 2 in the manuscript itself. As a result, the majority of the supplemental data has been added to the manuscript itself. Specifically, S1 Table, S2 Table, and S3 Table have been converted into Table 2, Table 4, and Table 5 respectively.**

**Additionally, we agree that donor age is a very important prognostic factor that should be included in our analysis of post-transplant mortality. As a result, we added donor age to every multivariate regression examining post-transplant mortality. These changes can be found in Table 3 and in the 2^nd^ and 3^rd^ sub-sections of the results sections.**

Reviewer 2:

1. Thank you for having partly answered to my questions. However, I notice that the answers have been provided in the supplementary material section.

**Author Response: Thank you for these important recommendations. We agree that it is important to highlight the issues brought up by reviewer 2 in the manuscript itself. As a result, the majority of the supplemental data has been added to the manuscript itself. Specifically, S1 Table, S2 Table, and S3 Table have been converted into Table 2, Table 4, and Table 5 respectively.**
